# Are asymmetric designs of tibial components superior to their symmetric counterparts for constrained condylar total knee arthroplasty using metal block augmentation?

**DOI:** 10.1186/s42836-024-00277-9

**Published:** 2024-11-04

**Authors:** Ryosuke Kabu, Hidetoshi Tsushima, Yukio Akasaki, Shinya Kawahara, Satoshi Hamai, Yasuharu Nakashima

**Affiliations:** 1 Department of Orthopedic Surgery, Saga Ken Medical Centre Koseikan, Saga, 840-0861 Japan; 2 Department of Orthopedic Surgery, Saiseikai Yahata General Hospital, Fukuoka, 805-0050 Japan; 3 Department of Orthopedic Surgery, Kyushu University, Fukuoka, 812-8582 Japan

**Keywords:** Total knee arthroplasty, Asymmetric tibial components, Metal block augmentation, Bony coverage and fit, Revision total knee arthroplasty, CT-based simulation

## Abstract

**Purpose:**

In total knee arthroplasty (TKA), asymmetric tibial components have been developed alongside symmetric tibial components to enhance bony coverage at the tibia. In primary TKA and revision TKA for patients with significant bone defects, augmentation is employed to fill the bone defect. However, there have been no reports on bony coverage of the tibial component of the revision system in the cases of bone defects. Therefore, we simulated bone defects using CT and compared the bony coverage of asymmetric and symmetric tibial components in the revision TKA system.

**Methods:**

This study included 45 patients (50 knees involved) with medial osteoarthritis. Preoperative CT scans were used to simulate placement using ZedKnee. Three models were evaluated: Persona Revision PCCK (Zimmer) for the asymmetric component, NexGen LCCK (Zimmer) for the symmetric component, and the ATTUNE revision system (Depuy-Synthes). A 130-mm stem extension was utilized. Augmentations of each thickness were placed to simulate bone defects of 5, 10, and 15 mm. The coverage, overhang, and underhang rates were measured for each slice and compared among the models.

**Results:**

In terms of coverage, the rate was greater for PCCK at 0 mm, and only ATTUNE exhibited a significantly lower coverage at 5 and 10 mm. There was no significant difference in coverage at 15 mm. At 0 mm, PCCK demonstrated less posterior underhangs. At 5 and 10 mm, PCCK showed less anterior overhang but more anterior underhang. At 15 mm, PCCK had a less anterior overhang, with an overhang in the posterior region but less underhang. When overhang and underhang were combined and compared, the asymmetric component generally yielded superior results.

**Conclusion:**

In the cases of bone defects, asymmetric components demonstrated reduced anterior overhang and decreased posterior underhang, resulting in greater bone coverage. This may contribute to improved long-term outcomes in the revision TKA system.

## Introduction

Total knee arthroplasty (TKA) is a highly effective surgical procedure for relieving pain and restoring function in patients with severe joint destruction due to osteoarthritis. To improve the outcomes of TKA, several factors must be carefully considered, including alignment, ligament balance, implant positioning, and fixation methods. Among these factors, the bony coverage and fit of the implant are critical factors dictating long-term success [1–4]. Proper bony coverage and fit can prevent implant subsidence, loosening, and pain caused by contact with surrounding soft tissues [5–13].

Traditionally, symmetric tibial components have been used in primary TKA. However, recent advancements have led to the development and use of asymmetric designs, which are believed to provide better bony coverage of the tibial resection surface, resulting in improved outcomes [14]. Semi-constrained TKA systems, often employed in patients with poor ligament balance or significant bone defects, rely on metal augmentation to fill these defects in both primary and revision TKA. Achieving greater bony coverage is essential for metal block augmentation to improve implant stability and long-term outcomes.

Despite the recognized importance of bony coverage in TKA, there is a lack of reports specifically addressing bony coverage in semi-constrained TKA systems with metal block augmentation and stem extension. Therefore, the purpose of this study was to compare the bone coverage and fit of asymmetric vs. symmetric tibial components in a semi-constrained TKA system using a CT-based simulation. By analyzing the differences in coverage, overhang, and underhang at different augmentation levels, we aimed to determine the optimal tibial component design for improving surgical outcomes in revision TKA.

## Materials and methods

### Patient selection

This was a retrospective comparative study with an evidence level of III after approval from the Institutional Review Board (No. 2020–204). All patients with osteoarthritis who underwent TKA were treated at the Department of Orthopedic Surgery at Kyushu University, Japan. The study involved 45 patients (5 males and 40 females) and 50 knees with medial knee osteoarthritis. Patients with inflammatory diseases, such as rheumatoid arthritis and lateral knee osteoarthritis, were excluded.

### Data acquisition

Preoperative computed tomography (CT) scan (Aquilion ONE: Canon Medical Systems Corporation, Tochigi, Japan) was performed before TKA. All CT scans of the ipsilateral lower extremity, ranging from the femoral head to the ankle joints, were taken with the same protocol, consisting of 2.0-mm-thick contiguous slices. The CT images were obtained in Digital Imaging and Communications in Medicine (DICOM) format. The data were imported to perform surgical simulations, such as placement of implants and calculation of parameters with ZedKnee software (LEXI, Tokyo, Japan) [15].

### Virtual surgery for implantation of the tibia

**Fig. 1:**
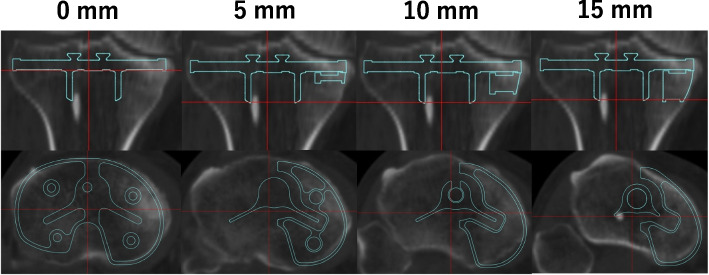
Medial block augmentations of 5 mm, 10 mm, and 15 mm were applied

Three tibial base plates and augmentations were evaluated: Design A: Persona CCK (Zimmer Biomet, Warsaw, IN, USA) as the asymmetric type and Design B: NexGen LCCK (Zimmer Biomet, Warsaw, IN, USA) and Design C: ATTUNE revision system (Depuy-Synthes, Warsaw, IN, USA) as the symmetric type. The resection level was set at 10 mm from the lateral tibial plateau for the primary bone cut, and the augmentation was set at 0 mm for the metal block. The rotational axis for tibial baseplate placement was defined by the Akagi line, which is drawn from the medial edge of the patellar tendon to the center of the posterior cruciate ligament (PCL) attachment [16]. An optimal fit was defined as less than 1 mm of overcoverage and less than 2 mm of undercoverage [17], with the optimal size determined by the component that satisfied these criteria on the lateral aspect of the tibia. The tibial base plates were fitted with a 135-mm cementless stem extension, ensuring that they were fully seated within the medullary canal. It is available for all the implants and offset adjustments were made as necessary. Offset correction was used in 49 out of 50 knees with PCCK, 47 with LCCK, and all 50 with ATTUNE. Medial block augmentations of 5 mm, 10 mm, and 15 mm were applied. The coverage of each augmentation level was assessed across transverse CT slices (Fig. 1).

### Image analysis

**Fig. 2:**
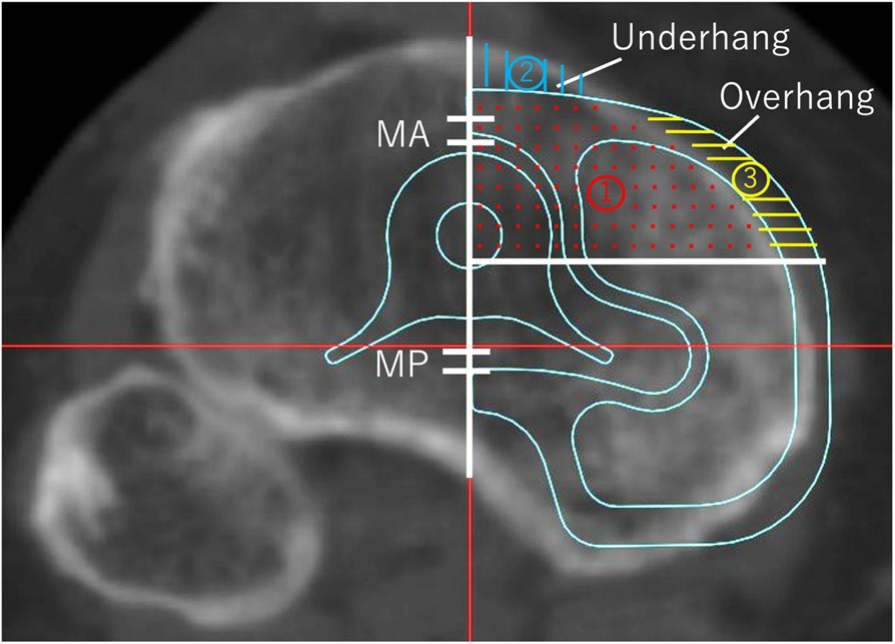
Import the image into ImageJ and take measurements. coverage(%): The proportion of component covering bone = ①/(① + ②) X 100. overhang(%): The proportion of component overhanging = ③/(① + ③) × 100. underhang(%): The proportion of bone not covered by the component = ②/(① + ②) × 100. We measured in the anterior (medial anterior area; MA) and posterior (medial posterior area; MP) regions, respectively

Similar to a previous study [18], the CT images were imported into ImageJ software (Public Domain, National Institute of Health, NIH, Bethesda, Maryland, USA) for quantitative analysis. The area of the ROI (region of interest) was measured using ImageJ. The following parameters were calculated (Fig. 2): Bony Coverage (%): the proportion of bone covered by the component, calculated as ①/(① + ②) × 100%. Overhang (%): the proportion of the component extending beyond the bone, calculated as ③/(① + ③) × 100%. Underhang (%): the proportion of bone not covered by the component, calculated as ②/(① + ②) × 100%. Measurements were performed at the anterior (medial anterior area; MA) and posterior (medial posterior area; MP) regions relative to the stem insertion site. To assess the overall fit, the combined values of overhang and underhang were calculated for each region and augmentation level. This provided a comprehensive measure of the implant's conformity to the bone surface. Good inter-rater reliability for all measurements was found between the two observers in terms of the intra-class correlation coefficient (ICC). The ICC for all measurements was above 0.9.

### Statistical analysis

Statistical comparisons between the different tibial components were made using Tukey’s honestly significant difference (HSD) test or the Steel–Dwass test to determine significant differences in measured parameters across the components and augmentation levels. A P value less than 0.05 was considered significant. All P values were two-sided. The statistical software JMP Pro version 14 (SAS, Cary, NC, USA) was used for all analyses.

## Results

**Fig. 3:**
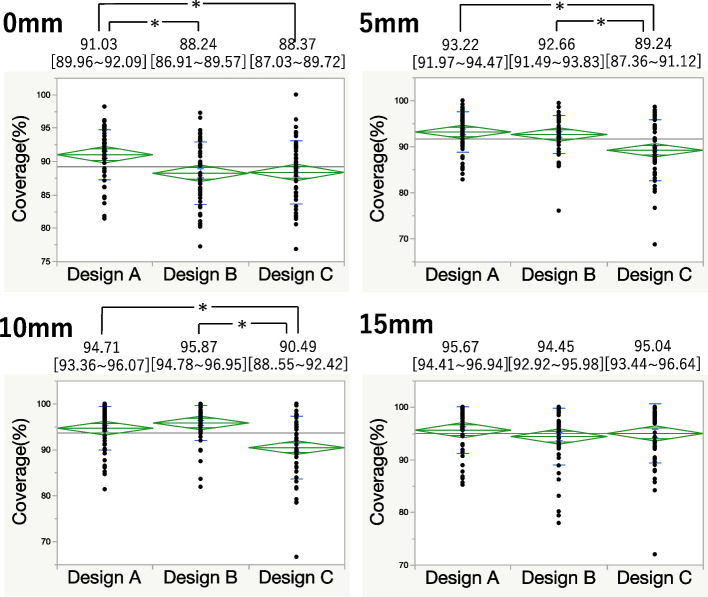
Comparison of coverage in each slice. (*P < 0.05)

At 0 mm metal augmentation, Design A exhibited significantly higher coverage than both Design B and Design C. There was no significant difference in coverage between Design A and Design B at the levels of 5 mm and 10 mm metal block augmentation. Design C demonstrated lower coverage at 5 mm and 10 mm augmentations than Design A and Design B. At 15 mm augmentation, no significant differences were found in coverage among the components (Fig. 3).

Design A showed the least posterior underhang and lowest combined overhang and underhang values.

**Fig. 4:**
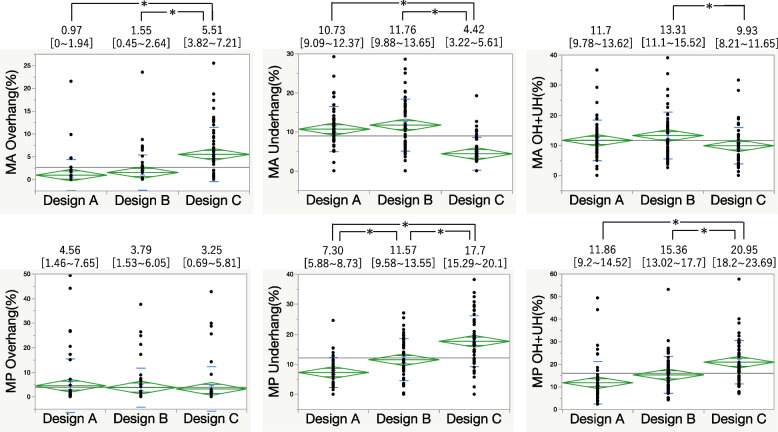
Comparison of the overhang (OH) and underhang (UH) at the 0 mm slice and the evaluation of the fit. (*P < 0.05)

**Fig. 5:**
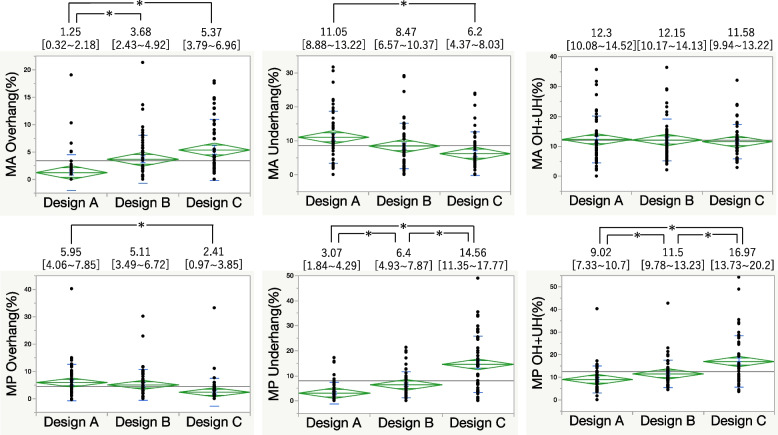
Comparison of the overhang (OH) and underhang (UH) at the 5 mm slice and the evaluation of the fit. (*P < 0.05)

**Fig. 6:**
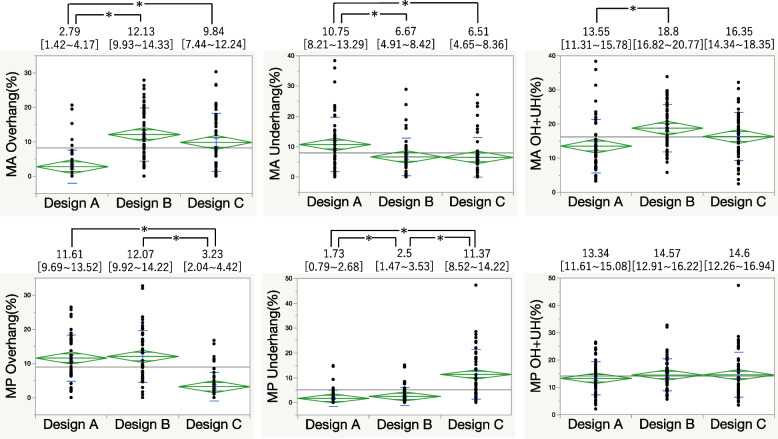
Comparison of the overhang (OH) and underhang (UH) at the 10 mm slice and the evaluation of fit. (*P < 0.05)

**Fig. 7:**
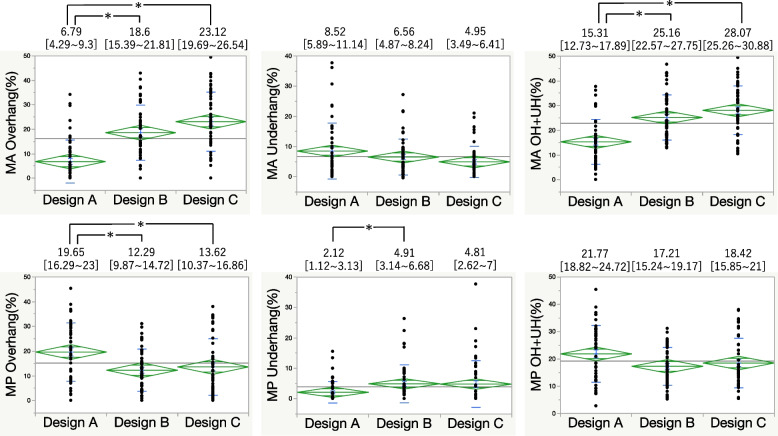
Comparison of the overhang (OH) and underhang (UH) at the 15 mm slice and the evaluation of fit. (*P < 0.05)

For the 0 mm and 5 mm metal block augmentation, Design A had the lowest anterior overhang but a higher underhang than Design C did in the MA area. At the MP area, PCCK had a significantly lower underhang. Combined overhang and underhang values indicated that Design A provided the best fit (Figs. 4 and 5). At 10 mm augmentation, Design A showed the least anterior overhang, while Design B exhibited anterior overhang and Design C had posterior overhang (Fig. 6). At 15 mm augmentation, Design A had the lowest anterior overhang and combined overhang and underhang values (Fig. 7).

## Discussion

The findings of this study, though conducted under CT simulation, demonstrated that asymmetric designs were generally superior in terms of the coverage and fit of metal block augmentation, which is often used to address bone defects. Our results showed that Design A, an asymmetric component, consistently provided superior bony coverage compared to the symmetric Design B and Design C. With both 0 mm and 5 mm of augmentation, Design A exhibited significantly greater coverage and lower combined overhang and underhang values, indicating a better fit to the bone surface.

In this study, at the osteotomy level without augmentation, the asymmetric design exhibited bone coverage superior to the symmetric tibial component. This outcome aligns with several prior studies regarding bone coverage in primary TKA [17]. At the 5 mm and 10 mm metal block augmentation levels, no significant difference in coverage was observed between the Design A and Design B groups. With Design A, significantly less overhang was noted in the anteromedial region, but this was offset by an increase in underhang. Consequently, there was no difference when overhang and underhang were combined, suggesting that suitability varied depending on the case.

Overhangs and underhangs have been clinically reported as issues. Past studies categorized an underhang as less than 3 mm and an overhang as more than 3 mm [19], with an overhang of more than 2 mm considered unacceptable [17]. In primary TKA, an overhang is known to cause irritation of the surrounding soft tissues, leading to reduced postoperative scores [5–9], while an underhang is associated with risks of implant subsidence and loosening [10–13]. Consequently, by aggregating overhang and underhang values, an overall assessment of fit showed that although significant variances existed, depending on the volume of bone loss, asymmetric components generally yielded superior results. In this instance, compared with other models, employing the symmetric Design C implant tended toward anterior placement when fitting the stem extension within the medullary cavity, resulting in diminished coverage and increased anterior overhang and posterior underhang. This study demonstrated that asymmetric components, relative to their symmetric counterparts, had reduced anterior overhang and posterior underhang, suggesting the potential for improved postoperative outcomes with asymmetric components. However, the impact of tibial component coverage in revision systems on clinical outcomes remains uncertain, underscoring the need for further evaluation in actual cases and clinical outcomes.

Accurate placement of the tibial component enhances knee kinematics, mitigates patellar complications, and improves functional outcomes [20, 21]. In TKA, both rotational alignment and bone coverage of the tibial component are crucial. Previous studies have demonstrated that coverage varies according to the rotational axis. The Insall line, extending from the center of the posterior cruciate ligament (PCL) insertion to the medial third of the tibial tuberosity, has been traditionally used to determine tibial implant rotation [19]. In this study, we utilized the Akagi line, which connects the medial edge of the patellar tendon to the PCL attachment and is regarded as a reproducible intraoperative indicator.

No definitive findings have been reported on how the fitness of metal block augmentation influences long-term outcomes. Metal block augmentation provides robust initial fixation and enables early weight bearing, making it particularly beneficial for elderly patients with comorbid conditions, where a shortened rehabilitation period is advantageous, and for patients with poor bone quality [22]. Additionally, block types with osteotomy perpendicular to the functional axis of the lower limb have demonstrated superior load-bearing capacity and outcomes [23]. Hamai et al. identified a radiolucent line beneath the metal block augmentation in 58% of patients; however, no enlargement or loosening was observed during a follow-up period of up to 6 years post-surgery [24]. Although there may be no significant difference in mid-term outcomes, the coverage provided by metal augmentation is crucial for long-term success.

Our study also highlighted the limitations of symmetric designs, particularly the ATTUNE revision system, which showed lower coverage and higher overhang and underhang values at various augmentation levels. These findings are consistent with other studies that have reported inferior fit and higher complication rates associated with symmetric tibial components in complex revision patients.

One of the strengths of this study is the use of CT-based simulation, which provides a detailed and accurate assessment of component fit and coverage. This approach allows for precise measurement of coverage, overhang, and underhang, facilitating a comprehensive comparison between different component designs. However, it is important to note that simulation studies have inherent limitations, and further clinical evaluation is necessary to validate these findings in actual surgical settings.

Future research should focus on long-term clinical outcomes associated with the use of asymmetric tibial components in revision TKA. Additionally, exploring the impact of different surgical techniques and patient-specific factors on implant fit and performance will be crucial for optimizing TKA outcomes.

This study has several limitations. First, the sample size of 45 patients (50 knees) may limit the generalizability of the results. Large-sized studies are needed to confirm these findings and ensure that they can be extrapolarted to a broader patient population. Second, for component selection, only three specific tibial components were analyzed. The results may not be directly applicable to other asymmetric or symmetric tibial components available on the market. Future studies should include a wider range of implant designs to provide more comprehensive comparisons. Third, there was a lack of clinical correlation. The study did not assess clinical outcomes, such as patient-reported satisfaction, functional scores, or long-term implant survival. While the simulation results are promising, clinical trials are necessary to correlate these findings with actual patient outcomes and to determine the true clinical significance of improved bony coverage and fit. Addressing these limitations in future research will be crucial to validate this study's findings and optimize implant selection and surgical techniques for improving the outcomes of revision TKA.

## Conclusion

In conclusion, this study provided compelling evidence that asymmetric tibial components offered superior bony coverage and fit in revision TKA compared to symmetric designs. These findings support the use of asymmetric components to improve surgical outcomes and patient satisfaction in patients receiving complex knee arthroplasties.

## Data Availability

All the data are available from the corresponding author on reasonable request.
